# On the Possibility of Using 5-Aminolevulinic Acid in the Light-Induced Destruction of Microorganisms

**DOI:** 10.3390/ijms25073590

**Published:** 2024-03-22

**Authors:** Anna Zdubek, Irena Maliszewska

**Affiliations:** Department of Organic and Medicinal Chemistry, Faculty of Chemistry, Wrocław University of Science and Technology, 50-370 Wrocław, Poland; anna.zdubek@pwr.edu.pl

**Keywords:** antimicrobial photodynamic inactivation, 5-aminolevulinic acid, pathogens, bacteria, eradication

## Abstract

Antimicrobial photodynamic inactivation (aPDI) is a method that specifically kills target cells by combining a photosensitizer and irradiation with light at the appropriate wavelength. The natural amino acid, 5-aminolevulinic acid (5-ALA), is the precursor of endogenous porphyrins in the heme biosynthesis pathway. This review summarizes the recent progress in understanding the biosynthetic pathways and regulatory mechanisms of 5-ALA synthesis in biological hosts. The effectiveness of 5-ALA-aPDI in destroying various groups of pathogens (viruses, fungi, yeasts, parasites) was presented, but greater attention was focused on the antibacterial activity of this technique. Finally, the clinical applications of 5-ALA in therapies using 5-ALA and visible light (treatment of ulcers and disinfection of dental canals) were described.

## 1. Introduction

It is clear that antibiotics have saved millions of lives, but it is also undeniable that many antibiotics are now less effective because microbes develop resistance to these chemicals. The causes of microbial resistance to antibiotics are diverse, but the main sources of its development are considered to be the excessive use of antibiotics in agriculture, the inappropriate use of antibiotics in medicine, and even the consumption of a more processed diet [1]. Understanding the mechanisms of antibiotic resistance and also searching for new antimicrobial substances are essential to solve this global health problem. However, these strategies seem to be insufficient, and it is necessary to search for new “non-antibiotic antibacterial technologies” that will complement or is an alternative to classic antibiotic therapy.

One technique that has gained a lot of popularity in recent years is photodynamic therapy; however, this method, in relation to the control of pathogenic microorganisms, is called antimicrobial photodynamic inactivation (aPDI). This technique is based on the light-induced overproduction of reactive oxygen species (ROS), which kills target microorganisms regardless of their resistance to conventional treatments [2]. 

### 1.1. Photodynamic Therapy

From antiquity to the present, light has been used as a therapeutic factor in medicine. Photodynamic therapy (PDT) originated in ancient Egypt, Greece, and India, where sunlight and various herbs were used to treat skin diseases. However, it was forgotten for many years, only to resurface at the beginning of the 20th century [3]. The modern date of the discovery of photodynamic therapy is considered to be 1900, when medical student Oscar Raab observed that the incubation of protozoa (*Paramecium* spp.) with acridine red and exposure to light (wavelength corresponding to the red color) leads to its death. In contrast, incubation of the protozoa with the dye without the interference of light has no effect on their viability. It has been established that the only variable parameter is light, and its amount determines the toxic effect. Subsequently, Professor Herman von Tappeiner for the first time used the definition of ‘photodynamic action’ and suggested the importance of oxygen in the whole process [4,5]. In 1978, PDT was clinically examined by Dougherty et al. [6]. They observed a complete or partial remission of subcutaneous or cutaneous malignant tumors. Hematoporphyrin derivative (HpD) was the chemical compound (photosensitizer) utilized in this treatment. It is worth mentioning that in 1999 the FDA approved PDT for the treatment of precancerous skin conditions of the face and scalp [6,7]. PDT has evolved since then, and its clinical use in medicine has expanded to other fields in addition to cancer treatment.

#### Mechanism of aPDI and the Possibility of Developing Tolerance

Antimicrobial photodynamic inactivation is a treatment method that combines three different components: visible light of a particular wavelength, photosensitizer (PS) at a non-toxic concentration, and molecular oxygen. The process starts with the absorption of light by the PS, which induces a chain of reactions that result in the formation of ROS. When exposed to a specific wavelength of light, the transition of the PS from the ground state to the excited singlet state can occur. Because the singlet state is highly unstable, electrons can lose energy and may return to the ground state through the emission of light (fluorescence) or production of heat. Subsequently, the PS can undergo the more stable triplet state through intersystem crossing. Then, the PS is able to react with biomolecules through two different mechanisms (type I and II). The products of type I and type II reactions are responsible for the induction of cell death [4,8].

The type I mechanism requires a transfer of the electron between the excited PS and the substrate (organic molecule), resulting in free radical production. Then, free radicals react with molecular oxygen, leading to the generation of ROS, including superoxide, hydrogen peroxide, and hydroxyl radicals [9,10].

On the other hand, the type II mechanism involves a reaction between PS and oxygen to generate singlet oxygen, which can then cause oxidation damage to the cell envelopes. Both of these mechanisms can occur in parallel, and the relationship between them is determined by a substrate of PS and oxygen concentration [10,11].

The resulting reactive molecules induce major changes in microbial cells, both in their morphology and in their functionality. The formation of ROS leads to a disruption of the cell envelopes and the deactivation of the membrane transport system. Functional changes in microbial cells mainly include a reduction in their enzymatic activity and inhibition of metabolic processes [12]. The most important advantage of aPDI that should be emphasized, is that it is not dependent on antibiotic resistance when used against bacterial pathogens.

It is believed that two main factors determine the success of this biocidal technique—the type of photosensitizer and light. Photosensitizers, which can be used successfully in photodynamic therapy for various types of cancer, have also been tested for their usefulness against pathogenic microorganisms. Other compounds with properties optimized for the killing of bacteria and fungi were synthesized [13]. For this reason, a wide range of photosensitizers are available and can be tested in clinical trials. These include natural chemicals such as curcumin, hypericin, and chlorophyllin, as well as classes of pigments such as phenothiazinium, porphyrins, chlorites, and phthalocyanines [14]. Excellent articles [15,16] provide a summary of data on light sources that can be used in aPDI.

In recent years, the development of bacterial resistance/tolerance to aPDI has been extensively studied, and a number of reviews on this phenomenon are available [17,18,19]. Although most authors reported that antimicrobial photodynamic therapy did not cause pathogen resistance to these eradication techniques, there is increasing evidence that resistance to photosensitizing drugs at low concentrations and tolerance to reactive oxygen species formed after exposure to light are possible [19].

**Figure 1:**
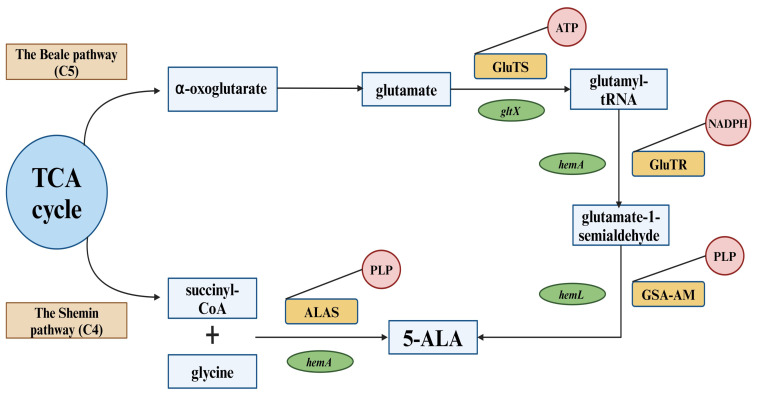
Biosynthetic pathways of 5-ALA. Key abbreviations are boxed. TCA cycle = the tricarboxylic acid cycle; ALAS = 5-ALA synthase; PLP = pyridoxal 5′-phosphate; GluTS = glutamyl-tRNA synthetase; GluTR = glutamyl-tRNA reductase; GSA-AM = glutamate-1-semialdehyde aminotransferase; ATP = adenosine triphosphate; NADPH = nicotinamide adenine dinucleotide phosphate; *hemA* = gene encoding 5-ALA synthase; *gltX* = gene encoding glutamyl-tRNA synthetase; *hemA* = gene encoding glutamyl-t-RNA reductase; *hemL* = gene encoding glutamate-1-semialdehyde aminotransferase.

This review aims to collect the main knowledge on 5-aminolevulinic acid (5-ALA) as a precursor to bacterial photosensitizers (porphyrins) (Figure 1), and to discuss the possibilities of using this amino acid in light-induced antimicrobial therapies. The synthesis of porphyrins using 5-ALA was first documented in 1953. Shemin et al. [20] discovered that “active” succinate and glycine in porphyrin synthesis can be substituted with 5-ALA. This implied that the source of all protoporphyrin atoms was 5-aminolevulinic acid.

## 2. Methods

The procedure to search the review of the literature on the application of 5-aminolevulinic acid included articles published between 1997 and 2023 (according to the Web of Science database). The database search was based on a logical formula of (5-aminolevulinic acid AND bacteria AND ((photodynamic AND therapy) OR (photodynamic AND inactivation))). This resulted in 115 articles, of which the abstracts were carefully analyzed to make an additional selection of the literature. The main objective was to find articles that present applications of 5-ALA in the photodynamic inactivation (photodynamic therapy) of bacterial pathogens. Publications divergent from the subject were excluded, and only papers written in English language were taken into consideration. In general, 65 publications met our criteria and were thoroughly analyzed.

Gram-positive bacteria represent a significantly higher number of research (64%) compared to Gram-negative bacteria (36%). This is probably due to the less-complex cell wall structure of Gram-positive bacteria, which influences the enhanced uptake of photosensitizer into the cell, resulting in an increase in the effectiveness of photo-eradication. Among all Gram-positive bacteria tested, *Staphylococcus aureus* was the most frequently selected research target (31%), while in Gram-negative bacteria it was *Klebsiella pneumoniae* (30%) and *Escherichia coli* (27%).

Some studies have examined not only the effect of 5-ALA on photosensitizing efficacy but also the effect of its derivatives such as methyl, hexyl esters, etc. This issue was also taken into account in this work.

## 3. Biosynthesis of 5-ALA

5-aminolevulinic acid, a non-proteinogenic amino acid, is one of the most essential components in the production of tetrapyrroles. This ketoacid is a common precursor in the biosynthesis of compounds including porphyrin, heme, vitamin B12, and chlorophyll [21,22]. 5-ALA has a considerable impact on cell development and metabolic flux. It is interesting that it can be synthesized by various forms of life, such as mammals, plants, fungi, and bacteria. Many studies have focused on alternative 5-ALA microbial production from renewable and affordable sources due to the difficult procedure and low yield of conventional chemical synthesis methods [23].

There are two known alternative routes in nature by which 5-ALA is generated, the C4 (Shemin pathway) and the C5 pathway, respectively [24] (Figure 1).

Some organisms produce 5-ALA by decarboxylative condensation of succinyl-CoA and glycine catalyzed by 5-ALA synthase (ALAS), known as the C4 pathway (Shemin pathway). Others produce 5-ALA along the C5 route from glutamate [25]. It is worth mentioning that mammals, fungi, and purple non-sulfur photosynthetic bacteria are the only organisms that use the C4 route [26]. On the contrary, in plants, archaea, and in most bacteria, the active pathway is the C5 route.

A brief overview of these two processes, as well as a description of how 5-ALA is synthesized, is provided below. To date, six chemical synthesizes of 5-ALA have been reported. Levulinic acid, 2-hydroxypyridine, furfural, furfurylamine, tetrahydrofurfurylamine, and succinic acid can all be used as substrates to synthesize 5-ALA [27]. With increased awareness of environmental protection, techniques that include the microbial production of 5-ALA have gained more attention than conventional chemical synthesis methods.

### 3.1. The Shemin Pathway (C4)

One of the two natural 5-ALA biosynthesis pathways is the C4 pathway. It was first described by David Shemin and his co-workers. Shemin and Rittenberg presented the first study on the incorporation of nitrogen atoms derived from glycine into heme in 1945. Shemin conducted research on his own heme, ingesting ^15^N-labelled glycine which resulted in the ^15^N-incorporation into his heme [28].

In 1952, David Shemin and Albert Neuberger laboratories identified succinyl-CoA as the second origin of carbon atoms for heme synthesis. It was possible by further investigation of incorporation using radioactively labelled acetate [29,30].

The research conducted by Shemin and independently by Neuberger also indicated that 5-ALA is formed from the condensation of succinyl-CoA with glycine [30,31,32]. The 5-ALA synthase enzyme (ALAS) (EC 2.3.1.37) mediates the C4 pathway with pyridoxal 5′-phosphate (PLP) as a cofactor. The reaction is initiated by the binding of glycine to PLP, which results in the formation of a Shiff base at the active site of the enzyme. The abstraction of a proton by a radical, followed by succinyl-CoA attachment, produces a transitional intermediate called 2-amino-3-ketoadipate. The removal of CoA and carbon dioxide then leads to the release of 5-ALA from the enzyme [33,34].

Astner et al. [35] presented the crystal structure of ALAS from *Rhodobacter capsulatus* for the first time. In addition, they compared the sequence of this enzyme derived from bacteria with that of human one. The sequences were 49% identical [35]. Knowledge of the crystal structure of this homodimeric enzyme allows for the modification of the Shemin biosynthesis pathway.

Over the past few years, significant effort has been invested to improve 5-ALA synthesis through the C4 route by performing ALAS overexpression. The main regulatory mechanism of the Shemin pathway is based on the engineering of the key enzyme ALAS, which is encoded by the *hemA* and *hemT* genes [36]. However, the *hemA* gene, which is mainly responsible for the activity of the synthase mentioned, has attracted the most attention from researchers. Tai et al. suggested for the first time that ALAS-encoded genes occur in each DNA sequence in *Escherichia coli* and *Rhodobacter sphaeroides*, and this was also confirmed by Neidle and coworkers [36,37]. Since then, there have been numerous articles on the microbial production of 5-ALA and the overexpression of ALAS. Unfortunately, 5-ALA microbial production is relatively inefficient [38]. Therefore, genetic engineering strategies to improve the microbial production yield of this chemical have become popular. For this purpose, synthetic bacterial strains, mainly *Escherichia coli* and *Corynebacterium glutamicum*, were initially used to produce 5-ALA [39].

The first report of ALAS gene overexpression was made in 1999 by Choi et al. [40]. They used the *hemA* gene, which encodes ALAS from *Bradyrhizobium japonicum*, and overexpressed it in the *Escherichia coli* strain (BL21(DE3)) with a yield of 2.62 g·L^−1^ and a productivity of 0.187 g·L^−1^·h^−1^. Similar studies were conducted in the following decades. The *hemA* gene of various microorganisms (*Rhodobacter sphaeroides*, *Rhodopseudomonas palustris*, *Agrobacterium radiobacter*, *Rhodobacter capsulatus*) and its overexpression in different strains of *Escherichia coli* strains (*Escherichia coli* BL21(DE3), *Escherichia coli* MG1655, *Escherichia coli* Rosetta (DE3)), remained the main focus of the research [40,41,42,43,44,45,46,47,48,49,50,51,52,53,54,55,56,57,58,59,60,61]. The most frequently used engineered strain was *Escherichia coli* BL21(DE3). However, to date, the maximum level of 5-ALA production for genetically engineered *Escherichia coli* (MG1655) is 30.7 g·L^−1^ with also the highest productivity of 1.02 g·L^−1^·h^−1^, which was demonstrated by Pu et al. [62].

In addition, an overexpression of the *hemA* gene derived from *Rhodobacter sphaeroides* in *Corynebacterium glutamicum* (ATCC 13032) cells was observed for the first time in 2016 by Lou et al. [63]. They obtained a yield of 7.53 g·L^−1^ with a productivity of 0.21 g·L^−1^· h^−1^. In the same year, another research group investigated that the overexpression of this gene, along with deletion of the *rhtA* gene and the *sucCD* gene, resulted in almost double the yield. To date, Wang et al. [64] have succeeded in achieving the highest 5-ALA production efficiency of 25.05 g·L^−1^ with a productivity of 0.52 g·L^−1^· h^−1^ by *Corynebacterium glutamicum*. The use of this pathogen for 5-ALA biosynthesis is still an area of extensive research [63,65,66,67,68].

Over the years, researchers have tried many techniques to achieve the maximum possible yield of 5-ALA synthesis through the Shemin pathway. They have employed not only the overexpression of the ALAS encoding gene, but additionally the simultaneous overexpression of numerous genes, as well as the deletion of certain genes, fermentation methods, and increased production with the use of D-glucose [42,48,65]. It can be concluded that further research and optimization of bioproduction methods will result in significantly higher yields of 5-ALA production through the C4 route.

### 3.2. The C5 Pathway (The Beale Pathway)

Another route of 5-ALA biosynthesis that occurs in nature is the C5 pathway. Originally, 5-ALA was first identified in 1953 in duck blood [20]. On the contrary, in 1970, Beale discovered 5-ALA in *Chlorella vulgaris* after adding levulinic acid (ALA dehydratase competitive inhibitor) in culture medium [69]. The C5 route occurs mainly in many bacteria, algae, and higher plants. This pathway utilizes glutamate generated from α-oxoglutarate by the TCA cycle as a substrate [21,22]. Glutamate is then converted to 5-ALA in a number of enzymatic reactions. The first product in this pathway formed by ligation of tRNA and glutamate is L-glutamyl-tRNA. This reaction is catalyzed by glutamyl-tRNA synthetase (GluTS). Subsequently, the reduction performed by glutamyl-tRNA reductase (GluTR) of the carboxyl group of the newly formed complex results in the formation of a formyl group (GluTR requires NADPH for its activity). Then L-Glu-tRNA can be converted to L-glutamic acid 1-semialdehyde (GSA). Eventually, 5-ALA is produced by the transamination of GSA by glutamate-1-semialdehyde aminotransferase (GSA-AM) [67,68,69,70,71,72]. This enzyme, like ALAS found in the C4 pathway, requires binding to pyridoxamine to produce 5′-diaminovalerate, which when rearranged, leads to the formation of 5-ALA [73,74].

The C5 pathway includes two main enzymes, GluTR (which is encoded by the *hemA* gene) and GSA-AM (which is encoded by the *hemL* gene). The overexpression of genes encoding these enzymes can affect the efficiency of 5-ALA production by microorganisms. Kang et al. [75] created a metabolic approach to produce 5-ALA through the C5 pathway in recombinant *Escherichia coli*. For this purpose, they used the GluTR encoder gene from *Salmonella arizonae*. The overexpression of this gene significantly increased 5-ALA production yield from 31.1 to 176 mg·L^−1^. Furthermore, a synergistic effect on 5-ALA production (2052 mg·L^−1^) was observed between *hemA* from *Salmonella arizonae* and GSA-AM from *Escherichia coli*. Production efficiency was also enhanced by the identification of the *rhtA* gene, which in *Escherichia coli* encodes a threonine/homoserine exporter. This enabled the creation of the *Escherichia coli* DALA construct, thus increasing the yield to 4.13 g·L^−1^ [75].

*Corynebacterium glutamicum*, comparable to the C4 route, can also be used to create 5-ALA in the C5 pathway. Ramzi et al. [76] observed for the first time an increase in 5-ALA production (2.2 g·L^−1^) in the C5 pathway by *Corynebacterium glutamicum* [76] as a result of co-overexpression of the *hemL* and *hemA* genes. Subsequently, Ko et al. [77] improved the results with an co-overexpression of *hemA^M^*, *hemL*, *rhtA*, and *odhI^T14A/T15A^*, with a yield of 2.9 g·L^−1^. To date, Zhang et al. have obtained the highest efficiency of 5-ALA production through the C5 pathway of the engineered *Corynebacterium glutamicum.* Zhang et al. have obtained a yield of 3.16 g·L^−1^ [78].

The bioproduction of 5-ALA by *Escherichia coli* and *Corynebacterium glutamicum engineered strains* through the C5 pathway is undeniably more challenging than through the C4 route [25,75,76,77,78,79,80,81,82,83,84,85,86,87,88]. It is probably related to the increase in the number of enzymatic steps involved compared to the Shemin pathway. As a result, production optimization is more complex and inefficient, according to data from the literature.

## 4. 5-ALA in Antimicrobial Photodynamic Inactivation

It is well known that 5-ALA is not an active photosensitizing agent. This amino acid is a universal precursor for the biosynthesis of various porphyrins, which can then be converted to heme in enzymatic processes. For a long time, it was believed that there was a common route of heme synthesis in all living organisms. This principle still applies to eucaryotes, but biochemical and bioinformatic evidence that emerged in the 1990s showed that there is more than one heme pathway in bacteria and archaea.

**Figure 2:**
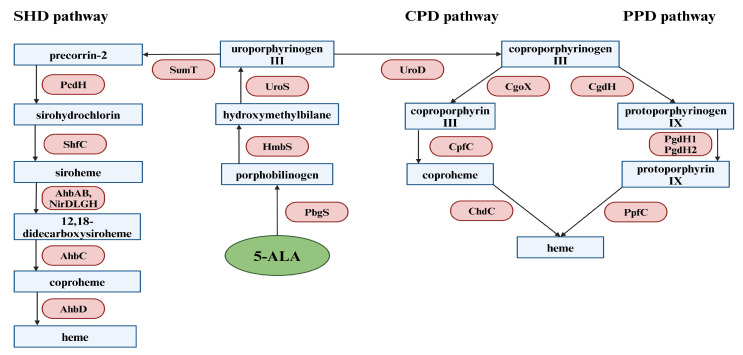
Schematic diagram of the metabolism of porphyrins produced by 5-ALA. Key abbreviations are boxed. UroS = uroporphyrinogen III synthase; HmbS = hydroxymethylbilane synthase; PbgS = porphobilinogen synthase; UroD = uroporphyrinogen III decarboxylase; CgdH = coproporphyrinogen III dehydrogenase; PgdH1 = protoporphyrinogen IX dehydrogenase 1; PgdH2 = protoporphyrinogen dehydrogenase 2; PpfC = protoporphyrin IX ferrochelatase; CgoX = coproporphyrinogen III oxidase; CpfC = coproporphyrin III ferrochelatase; ChdC = coproheme decarboxylase; SumT = uroporphyrinogen III methyltransferase; PcdH = precorrin-2 dehydrogenase; ShfC = sirohydrochlorin ferrochelatase; AhbAB = siroheme decarboxylase; NirDLGH = siroheme decarboxylase; AhbC = 12,18-didecarboxysiroheme decarboxylase; AhbD = coproheme decarboxylase. 5-ALA metabolism consists of three cytoplasmic reactions that generate uroporphyrinogen III. Further conversion of uroporphyrinogen III can occur in three different routes. The first pathway (CPD) occurs mainly in Gram-positive bacteria. The second pathway, called protoporphyrin dependent (PPD), is found in both eukaryotes and Gram-negative bacteria. The third route, which is called siroheme-dependent (SHD), occurs in archea and sulfate-reducing bacteria [89].

Following 5-ALA production, this compound is further converted in several enzymatic reactions common to all organisms to uroporphyrinogen III, which is the substrate for all three heme biosynthesis pathways. The first reaction is the condensation of two 5-ALA molecules catalyzed by porphobilinogen synthase (PbgS). This results in the formation of porphobilinogen (PBG). The next step involves hydroxymethylbilane synthase (HmbS), which catalyzes the synthesis of 1-hydroxymethylbilane (HMB) by combining four porphobilinogen molecules. Then, HMB undergoes cyclization through uroporphyrinogen III synthase, eventually leading to the formation of uroporphyrinogen III [89]. As can be seen in Figure 2, the heme biosynthetic pathways branch at the common intermediate uroporphyrinogen III and are named according to their corresponding key intermediates [89].

Two routes of heme biosynthesis have been described, which involve porphyrin intermediates and proceed through the coproporphyrinogen III branch. The first “protoporphyrin-dependent” pathway was called PPD and was found to be characteristic of eukaryotes and many Gram-negative bacteria. The PPD pathway, one of the three heme biosynthesis pathways, was the first to be identified. All of the enzymes involved in this route have already been adequately characterized, considerably facilitating our understanding of the catalytic mechanism of the PPD route. There are several enzymatic reactions involved in the conversion of uroporphyrinogen III to heme. The first enzyme that participates in the process is uroporphyrinogen III decarboxylase (UroD). Its action involves the decarboxylation of the uroporphyrinogen III side chains to methyl groups, resulting in the formation of coproporphyrinogen III. The following step includes the conversion of propionate groups to vinyl substituents by bacterial coproporphyringen III dehydrogenase (CgdH), which leads to the transformation of coproporphyrinogen III into protoporphyrinogen IX. The subsequent stage requires the alteration of protoporphyrinogen IX to protoporphyrin IX using special bacterial dehydrogenases. Most Gram-negative bacteria have one of the two dehydrogenases involved in this step. The first dehydrogenase is the oxygen-independent PgdH1. It participates in the coupling of protoporphyrinogen IX oxidation to anaerobic respiratory chains. The second dehydrogenase is PgdH2, which activity has not yet been defined in detail. In the final step of the PPD pathway, ferrous iron is incorporated into the protoporphyrin IX skeleton, resulting in the generation of heme. The enzyme that catalyzes this reaction is protoporphyrin IX ferrochelatase (PpfC) [89].

The second “coproporphyrinogen-dependent” pathway is called the CPD pathway and occurs mainly in Gram-positive bacteria. The first stage of the CPD route is identical to the PPD pathway and includes the application of the UroD enzyme. The next process in this pathway is the oxidation of coproporphyrinogen III to coproporphyrin III by coproporphyrinogen oxidase (CgoX). The penultimate step involves the insertion of the ferrous ion into the coproporphyrin III skeleton through the coproporphyrin III ferrochelatase (CpfC), leading to the formation of coproheme. Eventually, coproheme is converted to heme using coproheme decarboxylase (ChdC) [89].

The third metabolic pathway (dependent on precorrin-2) is called the “siroheme-dependent” pathway (SHD). This route has been discovered in the cells of sulfate-reducing bacteria and archaea, and it is known as the most ancient one. The first enzyme involved in the SHD pathway is SumT (S-adenosyl-L-methionine-dependent uroporphyrinogen III methyltransferase), which catalyzes the conversion of uroporphyrinogen III to precorrin-2. Subsequently, precorrin-2 is transformed through precorrin-2 dehydrogenase (PcdH) into sirohydrochlorin, and then sirohydrochlorin ferrochelatase (ShfC) makes an insertion of a ferrous ion into the compound skeleton, generating siroheme. The next stage requires siroheme decarboxylation to 12,18-didecarboxysiroheme by siroheme decarboxylases (AhbAB, NirDLGH). The penultimate stage includes comproheme formation, which is promoted by AhbC. Finally, heme is synthesized through oxidative decarboxylation from coproheme with the use of AhbD [89].

**Figure 3:**
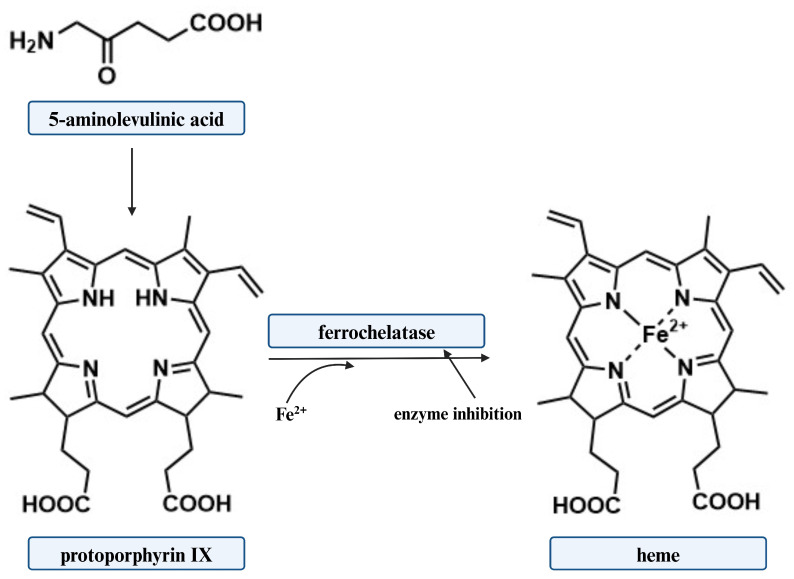
Scheme of conversion of 5-ALA to protoporphyrin IX and protoporphyrin IX to heme using ferrochelatase enzyme.

It is widely accepted that the effectiveness of 5-ALA-based aPDI as a photosensitizer precursor in light-induced inactivation of Gram-negative bacteria depends on the intracellular concentration of protoporphyrin IX (PpIX). There are several strategies to increase the concentration of these molecules in bacterial cells. It is possible to achieve this by controlling the activity of ferrochelatase, the enzyme that catalyzes the conversion of PpIX to heme (Figure 3). This strategy is based on the inhibition of enzyme activity, leading to the accumulation of PpIX in cells. Several inhibitors of ferrochelatase activity have been identified [90,91].

Sishtla et al. [92] described that nearly 20,000 compounds from ChemDiv and ChemBridge libraries were evaluated on a high-throughput screen to assess their effect at 10 µM on ferrochelatase activity. It turned out that only 93 structures (0.46%) reduced the activity of this enzyme by more than 50%. Interestingly, 62 compounds had a triazolopyrimidinone skeleton.

On the other hand, exogenous administration of 5-ALA can also improve the production and accumulation of PpIX. Nevertheless, both methods still need to be thoroughly investigated [93,94].

Following the metabolic patterns of heme synthesis, it should be recognized that the effectiveness of photodynamic inactivation of Gram-positive bacteria and eukaryotic cells (fungi, yeast) based on 5-ALA should be dependent on the level of intracellular coproporphyrin. It seems, however, that this view of the problem is simplified. A few years ago, very interesting results were presented by Nitzan et al. [95]. These authors described that the difference in the rate of bacterial photo-inactivation when using 5-ALA as a photosensitizer precursor resulted from the distribution and amount of different porphyrins in the bacterial strains. The dominant porphyrin in staphylococcal strains was found to be coproporphyrin (68.3–74.6%). In *Bacillus cereus* (Gram positive), the dominant porphyrin was uroporphyrin (75.8%). On the other hand, porphyrin was not dominant in Gram-negative strains and the porphyrins found were mainly 5-carboxyporphyrin, uroporphyrin, 7-carboxyporphyrin, coproporphyrin, and protoporphyrin. The total production of porphyrin in Gram-negative bacteria was higher than in *Staphylococcus* strains, with the amount of coproporphyrin produced by cocci being 2 to 3 times higher than in Gram-negative strains.

Despite extensive research in this area, the detailed mechanism of 5-ALA-based aPDI is still debated. A commonly accepted hypothesis is that endogenous chromophores are excited by blue light, leading to energy transfer and the production of reactive oxygen species (ROS), primarily singlet oxygen (^1^O_2_) [96]. Highly cytotoxic ROS can attack and oxidize various biomolecules near them, including DNA, RNA, proteins, and lipids [97,98].

Cruz-Oliveira et al. [99] described the light-induced activities of protoporphyrin IX (PPIX), Zn-protoporphyrin IX (ZnPPIX), and mesoporphyrin IX (MPIX) against vesicular stomatitis virus (VSV) and evaluated the mechanisms involved in this biocidal activity. It was shown that incubation of the virus with sodium azide and α-tocopherol partially protected VSV from light-induced inactivation in the presence of porphyrins, suggesting that singlet oxygen (^1^O_2_) was the main reactive oxygen species produced by these molecules (referred to as a type II reaction). Furthermore, ^1^O_2_ was detected by 9,10-dimethylanthracene oxidation in photo-activated porphyrin samples, which confirmed this hypothesis.

On the other hand, some researchers believe that (^1^O_2_) radicals can generate only partial photo-destructive effects against both Gram-negative bacteria and staphylococcal cells [95].

As mentioned above, some authors have drawn attention to the possibility of bacterial tolerance toward the light-induced destruction of pathogens. It should be emphasized that these considerations do not apply to photo-eradication based on exogenous administration of 5-aminolevulinic acid as a precursor of natural photosensitizer. However, we believe that the antimicrobial blue-light technique (aBL) should be considered in this context. This method is based on the use of blue light (400–470 nm) to destroy pathogens, and it has been shown to be an effective technique “free from exogenous photosensitizers”, causing a strong bactericidal effect. The hypothesis of aBL-mediated killing is based on the photoexcitation of endogenous porphyrins, which results in the production of intracellular reactive oxygen species (ROS), which causes cell membrane damage, DNA damage, lipid peroxidation, etc. [100].

A decade ago, Guffey et al. [101,102] suggested that *Staphylococcus aureus* may adapt to blue light irradiation. Subsequent applications of blue light (405 nm) to *Staphylococcus aureus* (ATCC 25923) were shown to be increasingly useful over four cycles; however, starting from the fifth cycle, the effectiveness of photo-inactivation of these bacteria decreased significantly.

Amin et al. [103] reported that *Pseudomonas aeruginosa* showed a reduced susceptibility to sublethal aBL treatment after nine cycles of photo-inactivation. The number of cells that survived photo-eradication increased by approximately 2 log_10_ units compared to the first cycle. The authors did not consider these results to be a possibility of resistance emergence, but this phenomenon could indicate the development of tolerance.

Rapacka-Zdonczyk et al. [104,105] assessed the development of a stable tolerance of Gram-positive bacteria (*Staphylococcus aureus*, *Enterococcus faecium*, and *Streptococcus agalactiae*), and Gram-negative bacteria (*Escherichia coli*, *Klebsiella pneumoniae* and *Pseudomonas aeruginosa*) to aBL. These authors observed a significant reduction in the antimicrobial effectiveness of aBL against all tested bacteria, and clearly demonstrated that the development of microbial tolerance to photodynamic inactivation is possible. Furthermore, studies have been performed with *Escherichia coli* K-12 mutants deficient in the *tolC*, *tolA*, *umuD,* and *recA* genes and some possible mechanisms for the development of tolerance to aBL tolerance have been revealed [105].

At this stage of the research, it is difficult to clearly identify the genetic elements that underlie the increased tolerance of bacteria to aBL (but also to aPDI), and various mechanisms may be involved in the response to photosensitization.

### 4.1. Strategies to Improve the Effectiveness of 5-ALA

A serious problem with the use of 5-ALA as a photosensitizer precursor is the chemical instability of this amino acid. The instability of ALA under neutral and basic conditions has been studied in detail, and it has been shown that this compound can readily dimerize to produce 2,5-dicarboxyethyl-3,6-dihydropyrazine (DHPY), which can spontaneously oxidize to 2,5-dicarboxyethylpyrazine [106,107]. Other 5-ALA transformations have also been proposed, involving the formation of porphobilinogen (PBG) and pseudoporphobilinogen [107,108,109].

Furthermore, 5-ALA is a zwitterion at physiological pH. It is known to be a hindered hydrophilic ion that is characterized by difficulties in crossing biological barriers and reaching the appropriate organelles in the cytoplasm [110]. Although most of the data on this problem relate to cancer cells, the delivery of this prodrug to the target site also applies to bacterial cells.

Various chemicals have been studied for topical administration of 5-ALA to improve penetration of this amino acid, including dimethylsulfoxide (DMSO) [111], 1-[2-(decylthio)-ethyl]azacyclopentan-2-one (HPE-101) [112], glyceryl monooleate [113], 6-ketocholestanol [114], and oleic acid [115].

**Figure 4:**
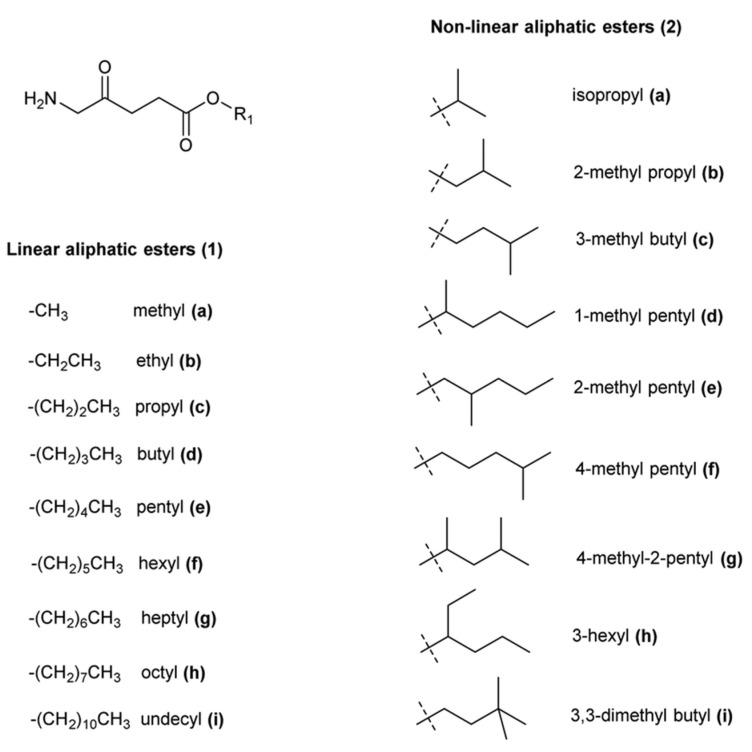
Aliphatic ester prodrugs of 5-ALA with a linear (1) or branched (2) alkyl component (taken from [117]). The letters (a–i) indicate the numbers of esters described by the authors. This article is licensed under a Creative Commons Attribution-NonCommercial 3.0 Unported License.

Therefore, it was reasonable to design 5-ALA derivatives that selectively target specific cell types or that release this prodrug only in the presence of appropriate enzymatic activity. The most effective approach to this problem appears to have been the synthesis of 5-aminolevulinic acid esters (Figure 4), and it should be emphasized that the resulting methyl ester (Metvix or MAL) has been approved for the treatment of some forms of cancer. On the contrary, hexyl ester has been approved for fluorescent bladder diagnostics as Hexvix (now Cysview) [116]. These clinically approved prodrugs and many other ALA esters have increased lipophilicity compared to 5-ALA alone, resulting in better penetration of cell envelopes and more effective accumulation of PpIX by various cells [117].

Until now, only a few studies have been conducted on the effectiveness of aPDI using 5-ALA derivatives. A comparison of the ability of 5-ALA and its methyl ester derivative (MAL) to photo-eradicate planktonic cultures and the biofilm formed by *Klebsiella pneumoniae* was carried out by Liu et al. [118]. These authors described that in the presence of white light, 5-ALA or MAL, planktonic cells, and biofilms were effectively inactivated. The mechanisms of the photo-toxic action of these prodrugs were also examined, and it was demonstrated that in the presence of 5-ALA or MAL, cleavage of genomic DNA and the rapid release of intracellular biopolymers were induced. Intensely denatured cytoplasmic contents and aggregated ribosomes were also detected by transmission electron microscopy.

In another study [119] the effectiveness of n-alkyl esters in the photodynamic destruction of some bacteria was examined. The methyl and butyl esters were the most effective for the photo-inactivation of bacterial pathogens. The authors proved that the effectiveness of bacterial destruction was a function of the total amount of porphyrins produced, regardless of their nature.

Many 5-ALA prodrugs based on alicyclic or substituted benzyl esters have also been synthesized, and extracellular delivery of these 5-ALA derivatives was found to result in increased levels of PpIX [120,121]. None of these derivatives have received clinical acceptance. The effective use of 5-ALA n-alkyl and benzyl esters in the treatment of acne caused by *Propionibacterium acnes* was shown in a patent [122]. More examples of the use of 5-ALA derivatives in aPDI are shown in Table 1.

The esterification of 5-ALA with various vitamins, monosaccharides, and nucleoside derivatives was investigated to improve the transport of this amino acid into pathogenic cells. Some 5-ALA ester conjugates with α-tocopherol (vitamin E) or vitamin D3 were synthesized, and it was confirmed that these systems of ALA ester derivatives with hydrophobic vitamins facilitated prodrug transport into cells and effectively increased the level of accumulated PpIX [123]. Regioselective acylation of 5-ALA with galactose and mannose has been reported to improve the selective accumulation of this amino acid in cells, and an evaluation of these prodrugs in human colon, breast, and lung cancer cells and angiogenic endothelial cells revealed that PpIX production was similar to ALA itself, while in normal fibroblasts the production of PpIX from the prodrugs was very low [124].

ALA esters with potential targeting properties have also been synthesized by esterifying the hydroxyl functions of nucleoside derivatives [125]. Interestingly, PpIX production was not correlated with the amount of 5-ALA delivered to cells of various cancer lines (human colon (SW480), breast (MCF7), lung (A549), ovary (A2780), cervix (HeLa)).

Analysis of data from the literature showed that several ALA peptide prodrugs were also obtained [126,127,128,129,130,131], and their ability to overcome cellular barriers and their impact on effective accumulation in PpIX were evaluated. These prodrugs were stable at physiological pH, non-toxic, and effectively penetrated the cytoplasmic membranes. To the best of our knowledge, the 5-ALA derivatives described above have not been examined for photo-inactivation of bacterial pathogens.

The rapid development of nanotechnology provides a new drug delivery characterized by exceptional selectivity, well-controlled drug release, and good biocompatibility [132]. Cyclodextrins, which are well-known nanocarriers of biological origin, exhibited many hydroxyl functions that enable the attachment of drug units, and these structures were used for the attachment of 5-ALA [133]. The application of other multivalent drug carriers such as dendrimers has been discussed, and this approach has been extensively explored as a strategy to overcome the poor systemic bioavailability of this prodrug [134,135]. Notable examples include the polymer encapsulation of ALA [136], gold nanoparticle-containing systems [137], and even ALA–squalene nanoassemblies have been obtained [138].

A very important issue in the latest nanotechnology is stimuli-responsive nanomedicines, that is, agents that can recognize the microenvironment in living systems and release therapeutic molecules to target tissues without affecting normal areas [139]. These drug delivery systems can control drug distribution, reduce potential side effects, and enhance therapeutic effectiveness. Response signals include endogenous stimuli such as low pH, glutathione concentration, and an increased expression of certain enzymes, as well as exogenous stimuli such as light, temperature, ultrasound, and chemicals. An excellent review summarizing the latest stimuli-responsive nanomedicines based on 5-ALA as a prodrug was published in 2023 [140]. In summary, it appears that none of the described nanomedicine strategies have been investigated for their application in aPDI.

As mentioned previously, the transformation of PpIX to heme in cancer cells is very limited, and this porphyrin can be efficiently accumulated. Unfortunately, PpIX in bacterial cells is further converted to heme by ferrochelatase, making its use in the photo-destruction of microorganisms limited. The maintenance of constant levels of porphyrins in the cell can be achieved by using chelating compounds. The chelation of iron ions will inhibit ferrochelatase activity, while increasing the level of PpIX in the cell, as its conversion to heme will not be possible [141,142].

**Table 1:** Applications of 5-ALA derivatives—mediated PDI against Gram-negative and Gram-positive bacteria.

Bacterial Strain	Concentration of 5-ALA Derivative	Light Dose	Light Source	Pre-Incubation Time with Derivative	Viability Reduction	Ref.
*Klebsiella pneumoniae* (ESBL-producing clinical isolate) planktonic	10.00 mM ALA-methyl ester	360 Jcm^−2^	150 W xenon lamp A wavelength range between 400 and 780 nm	4 h	4.52 log_10_ of CFUcm^−3^	[118]
*Klebsiella pneumoniae* (ESBL-producing clinical isolate) biofilm	10.00 mM ALA-methyl ester	360 Jcm^−2^	150 W xenon lamp A wavelength range between 400 and 780 nm	4 h	3.91 log_10_ of CFUcm^−3^	[118]
*Klebsiella pneumoniae* (non-ESBL-producing clinical isolate) planktonic	10.00 mM ALA-methyl ester	360 Jcm^−2^	150 W xenon lamp A wavelength range between 400 and 780 nm	4 h	4.32 log_10_ of CFUcm^−3^	[118]
*Klebsiella pneumoniae* (non-ESBL-producing clinical isolate) biofilm	10.00 mM ALA-methyl ester	360 Jcm^−2^	150 W xenon lamp A wavelength range between 400 and 780 nm	4 h	3.49 log_10_ of CFUcm^−3^	[118]
*Klebsiella pneumoniae* ATCC 700603 planktonic	10.00 mM ALA-methyl ester	360 Jcm^−2^	150 W xenon lamp A wavelength range between 400 and 780 nm	4 h	4.80 log_10_ of CFUcm^−3^	[118]
*Klebsiella pneumoniae* ATCC 700603 biofilm	10.00 mM ALA-methyl ester	360 Jcm^−2^	150 W xenon lamp A wavelength range between 400 and 780 nm	4 h	4.25 log_10_ of CFUcm^−3^	[118]
*Staphylococcus aureus* ATCC 6538 planktonic	3.90 µM ALA-methyl ester	20 Jcm^−2^	Laser light 600 nm	0.5 h	Almost complete inactivation	[143]
*Staphylococcus aureus* ATCC 6538 planktonic	3.90 µM ALA-hexyl ester	20 Jcm^−2^	Laser light 600 nm	0.5 h	Almost complete inactivation	[143]
*Escherichia coli* ATCC 35218 planktonic	3.90 µM ALA-methyl ester	20 Jcm^−2^	Laser light 600 nm	0.5 h	Almost complete inactivation	[143]
*Escherichia coli* ATCC 35218 planktonic	3.90 µM ALA-hexyl ester	20 Jcm^−2^	Laser light 600 nm	0.5 h	Almost complete inactivation	[143]
*Escherichia coli* K-12 planktonic	10.00 mM ALA-methyl ester	120 Jcm^−2^	400 W-halogen lamp	4 h	4.0 log_10_ of CFUcm^−3^	[119]
*Escherichia coli* K-12 planktonic	10.00 mM ALA-butyl ester	120 Jcm^−2^	400 W-halogen lamp	4 h	5.50 log_10_ of CFUcm^−3^	[119]
*Escherichia coli* Ti05 planktonic	10.00 mM ALA-methyl ester	120 Jcm^−2^	400 W-halogen lamp	4 h	5.00 log_10_ of CFUcm^−3^	[119]
*Pseudomonas aeruginosa* planktonic	10.00 mM ALA-methyl ester	120 Jcm^−2^	400 W-halogen lamp	4 h	2.00 log_10_ of CFUcm^−3^	[119]
*Staphylococcus aureus* planktonic	10.00 mM ALA-methyl ester	120 Jcm^−2^	400 W-halogen lamp	4 h	3.00 log_10_ of CFUcm^−3^	[119]

### 4.2. 5-ALA Mediated aPDI against Bacteria

It appears that there is considerable interest in using 5-aminolevulinic acid as a photosensitizer precursor for light-induced bacterial inactivation. There have been many articles on this topic showing the high effectiveness of this amino acid in aPDI against Gram-positive and Gram-negative bacteria [4,143]. As can be seen in Table 2 and Table 3, the biocidal activity of 5-ALA-based aPDI was studied against bacteria belonging to the following genera: *Enterococcus*, *Staphylococcus*, *Streptococcus*, *Corynebacterium*, *Mycobacterium*, and *Listeria*.

The results summarized in Table 2 show that the most attention was paid to the light-induced destruction of *Staphylococcus aureus*. It is well known that this pathogen leads to a variety of local infections, mainly of the skin and subcutaneous tissues, as well as a number of invasive diseases with a high mortality rate. Liu et al. [144] applied 5-ALA at a final concentration of 10 mM with a red light-emitting diode (633 nm, 288 Jcm^−2^) as a light source for the eradication of these cocci. The results obtained showed a reduction in viability of 5.22 log_10_ and 5.37 log_10_ for commercial strain and clinical isolate, respectively. Furthermore, they compared the effectiveness of treatment with the use of 5-ALA methyl ester (MAL) instead of 5-ALA. 5-ALA was found to be more efficient than its derivative [144].

The difficulty in combating staphylococcal strains is not only due to their resistance to antibiotics (MRSA), but also to their ability to form biofilms on both biotic and abiotic surfaces [145]. Researchers are interested not only in the problem of factors that influence the effectiveness of photodynamic inactivation of planktonic cells and biofilms using 5-ALA, but also in the connection between cell death and the amount of synthesized protoporphyrin. Zhang et al. [146] observed that after treatment (10 mM 5-ALA, 633 nm, 360 Jcm^−2^) over 99% of MRSA cells (methicillin-resistant *Staphylococcus aureus*) and MSSA (methicillin-sensitive *Staphylococcus aureus*) were killed in biofilm.

It should be mentioned that Huang et al. [147] reported the complete eradication of MRSA (planktonic) after 5-ALA treatment (at a concentration of 0.05 mM) and red light (633 nm, 384 Jcm^−2^).

The results obtained by Bohm et al. [148] indicated that aPDI with 5-ALA (at a final concentration of 2 mM) induced a reduction in approximately 6 log_10_ in planktonic (light doses ranged from 35 to 143 Jcm^−2^) as well as in biofilm cultures (energy fluence 143 Jcm^−2^) of *Staphylococcus aureus*.

Bae et al. [149] described the photo-inactivation of *Staphylococcus pseudintermedius*, which is responsible for skin infections in dogs, but there have also been reports of human infections, using 5-ALA and, interestingly, blue light. They also investigated the influence of blue light alone, which appeared to inhibit the growth of *Staphylococcus pseudintermedius* in a light-dose-dependent manner. However, its combination with 5-ALA increased the efficiency of aPDI with a maximum reduction in viability of 97.86% [149].

More examples of the use of 5-ALA-mediated aPDI toward Gram-positive bacteria are shown in Table 2.

**Table 2:** Applications of 5-ALA-mediated PDI against Gram-positive bacteria.

Bacterial Strain Gram (+)	Concentration of 5-ALA	Light Dose	Light Source	Pre-incubation Time with 5-ALA	Viability Reduction	Ref.
*Staphylococcus epidermidis* (clinical isolate) planktonic	1.00 mM	35 Jcm^−2^	ELH tungsten-halogen GE Quartzline lamps 25.2 mWcm^−2^	3 h	5 log_10_ of CFUcm^−3^	[148]
*Staphylococcus epidermidis* (clinical isolate) biofilm	1.00 mM	210 Jcm^−2^	ELH tungsten-halogen GE Quartzline lamps 25.2 mWcm^−2^	3 h	5 log_10_ of CFUcm^−3^	[148]
*Staphylococcus aureus* ATCC 25923 planktonic	2.00 mM	35 to 143 Jcm^−2^	ELH tungsten-halogen GE Quartzline lamps 25.2 mWcm^−2^	1 h	6 log_10_ of CFUcm^−3^	[148]
*Staphylococcus aureus* ATCC 25923 biofilm	2.00 mM	143 Jcm^−2^	ELH tungsten-halogen GE Quartzline lamps 25.2 mWcm^−2^	1 h	6 log_10_ of CFUcm^−3^	[148]
*Staphylococcus aureus* MRSA SA325 planktonic	0.05 mM	384 Jcm^−2^	Light-emitting diode 633 nm	4 h	Complete elimination	[147]
*Staphylococcus aureus* MRSA SA325 planktonic	10.00%	25 Jcm^−2^	Light-emitting diode 635 nm	3 h	2.05 log_10_ of CFUcm^−3^	[150]
*Staphylococcus aureus* MRSA (clinical isolate) biofilm	10.00 mM	360 Jcm^−2^	Light-emitting diode 633 nm	2 h	2.56 log_10_ of CFUcm^−3^	[146]
*Staphylococcus aureus* MSSA (clinical isolate) biofilm	10.00 mM	360 Jcm^−2^	Light-emitting diode 633 nm	2 h	2.71 log_10_ of CFUcm^−3^	[146]
*Staphylococcus pseudintermedius* (isolated from canine skin) planktonic	10.00%	55.2 Jcm^−2^	Light-emitting diode 465–470 nm	24 h	97.86%	[149]
*Corynebacterium jeikeium* K411 planktonic	1.00 mM	522 Jcm^−2^	Light-emitting diode 565 nm	-	4.5 log_10_ of CFUcm^−3^	[151]
*Mycobacterium abscessus* ATCC19977 planktonic	100.00 µg/mL	80 Jcm^−2^	Red light 585–635 nm	12 h	Approximately 50%	[152]
*Mycobacterium abscessus* ATCC19977 planktonic	100.00 µg/mL	160 Jcm^−2^	Red light 585–635 nm	12 h	Approximately 80%	[152]
*Streptococcus mutans* (isolate) biofilm	125.00 mM	-	Light-emitting diode 635 nm	30 s	2.23 log_10_ of CFUcm^−3^	[153]
*Streptococcus sobrinus* (isolate) biofilm	62.50 mM	-	Light-emitting diode 635 nm	30 s	2.87 log_10_ of CFUcm^−3^	[153]
*Enterococcus faecalis* (clinical isolate) planktonic	10.00 mM	288 Jcm^−2^	Light-emitting diode 633 nm	4 h	5.37 log_10_ of CFUcm^−3^	[144]
*Enterococcus faecalis* ATCC 51299 planktonic	10.00 mM	288 Jcm^−2^	Light-emitting diode 633 nm	4 h	5.22 log_10_ of CFUcm^−3^	[144]
*Listeria monocytogenes* ATCL3C 7644 planktonic	7.50 mM	18 Jcm^−2^	Light-emitting diode 400 nm	2 h	2.3 log_10_ of CFUcm^−3^	[154]
*Listeria monocytogenes* ATCL3C 7644 planktonic	10.00 mM	18 Jcm^−2^	Light-emitting diode 400 nm	2 h	3.7 log_10_ of CFUcm^−3^	[154]
*Listeria monocytogenes* ATCL3C 7644 biofilm	7.50 mM	18 Jcm^−2^	Light-emitting diode 400 nm	2 h	1.7 log_10_ of CFUcm^−3^	[154]
*Listeria monocytogenes* ATCL3C 7644 biofilm	10.00 mM	18 Jcm^−2^	Light-emitting diode 400 nm	2 h	3 log_10_ of CFUcm^−3^	[154]

Gram-negative bacteria are responsible for many life-threatening infections in humans, especially in the elderly, and may be resistant to the most commonly applied antibiotics, making the use of aPDI very important. It is known that Gram-negative bacteria are less susceptible to the phototoxic effects of light, and this property is attributed to the structure of their cell wall. These bacteria have a double lipid bilayer consisting of a peptidoglycan layer and an outer lipopolysaccharide layer, which results in a low degree of permeability to small lipophilic molecules.

The results presented in Table 3 show that most studies on the photo-destruction of Gram-negative rods were carried out using *Klebsiella pneumoniae.* This microorganism is found mainly in the intestinal tract. This opportunistic bacteria is responsible for a large number of nosocomial infections, including pneumonia, UTIs (urinary tract infections), and RTIs (respiratory tract infections) as well as community-acquired illnesses [155,156]. Antibiotic resistance remains a major challenge for infections caused by this pathogen. *Klebsiella pneumoniae* has considerably enhanced their defense systems over the past several years. The ability to produce β-lactamases (enzymes that hydrolyses β-lactam drugs) is especially responsible for the recent development of β-lactam antibiotic resistance. Some of the most common β-lactamases are ESBL (extended spectrum β-lactamase) [157]. This pathogen is characterized by a high tolerance to temperature [155].

Li et al. [158] presented an approach for controlling this microorganism with 5-ALA-aPDI. For this purpose, they used blue light (405 nm, 80 Jcm^−2^) and 5-ALA (at a final concentration of 50 µgmL^−1^). The treatment resulted in a 3 log_10_ reduction in planktonic culture. Furthermore, they observed that carvacrol preserves host cells in combined treatment, increasing the bactericidal effect at the same time [158].

Similar studies were conducted by Liu et al. [118]. These authors investigated the effect of 5-ALA (at a final concentration of 10 mM) and white light (360 Jcm^−2^) on the eradication of *Klebsiella pneumoniae* in both biofilm and planktonic culture. They conducted research on the ESBL-producing clinical isolate, non-ESBL-producing clinical isolate, and a commercial strain. Inactivation directed against the commercial strain resulted in a reduction in viability of 3.68 log_10_ and 3.09 log_10_ for planktonic culture and biofilm, respectively. Unfortunately, therapy for clinical isolates was slightly less efficient. A lower viability reduction was achieved for the non-ESBL-producing strain [118].

*Acinetobacter baumannii* is classified as a non-fermenting bacterium that is responsible mainly for 10% of all hospital-acquired infections caused by opportunistic Gram-negative microorganisms [159]. This pathogen is involved in skin and soft tissue infections and also contributes to UTIs. Infections of the blood and respiratory system, which can lead to death, have been identified as the most dangerous [160]. aPDI has been tested as an alternative method against *Acinetobacter baumannii* using different light sources and photosensitizers [161,162]. Studies conducted by Maliszewska et al. [163] showed that aPDI performed with the use of 5-ALA (at a final concentration of 1.25 mM) and two different light wavelengths is efficient against *Acinetobacter baumannii*. Exposure to blue light (405 nm) with an energy fluence of 32 Jcm^−2^ resulted in a lethal effect. On the other hand, irradiation with red light (635 nm) with an energy fluence of 102 Jcm^−2^ also enabled achieving the lethal effect. It is worth mentioning that they also tested the effectiveness of combined treatment. For this purpose, pentamidine was used at a final concentration of 75 nM to enhance the efficiency of 5-ALA-aPDI. The results obtained indicate that the addition of pentamidine allows one to achieve a lethal effect while reducing the energy fluence from 32 to 16 Jcm^−2^ and from 102 to 51 Jcm^−2^ for irradiation with blue and red light, respectively [163].

*Pseudomonas aeruginosa* is also a Gram-negative rod, leading to UTIs, ear infections, skin and soft tissue infections, and ventilator-associated pneumonia (VAP) with a high mortality rate [164,165]. It is pathogen resistant to many types of antibiotics, including β-lactams and aminoglycosides, as well as fluoroquinolones [166]. Photodynamic inactivation has also been investigated as a possible treatment for diseases caused by these bacteria. For example, Katayama et al. [167] demonstrated that the use of 5-ALA (at a final concentration of 0.50%) and blue light (410 nm, energy fluence of 9 Jcm^−2^) resulted in a reduction of 5 log_10_ in the viability of the biofilm.

Lee et al. [168] achieved complete photoinactivation of the *Pseudomonas aeruginosa* biofilm with 20 mM 5-ALA and red light (630 nm, energy fluence of 240 Jcm^−2^). The same results were obtained for the planktonic culture with slight modifications. Complete photoinactivation was achieved after treatment with a 10 mM or 7.5 mM 5-ALA with an energy fluence of 240 Jcm^−2^ or 360 Jcm^−2^, respectively [168].

Bohm et al. [148] received a 4 log_10_ reduction in planktonic culture with the use of 5-ALA (at a final concentration of 40 mM) and light with an energy fluence of 142 Jcm^−2^. However, *Pseudomonas aeruginosa* in the biofilm form did not respond to aPDI under the same treatment conditions [144].

Due to the increasing number of infections caused by *Enterobacter* spp. and the growing resistance to conventional treatments, aPDI has been studied as an alternative method of eradication of this pathogenic bacterium.

The study conducted by Bohm et al. [148] showed that *Escherichia coli* was not inactivated by 5-ALA-aPDI, both in planktonic and biofilm cultures [118]. For that purpose, they used 5-ALA at a concentration of 40 mM and light with an energy fluence of 142 Jcm^−2^.

The effect of PDI on *Escherichia coli* was also evaluated by Alqahtani et al. [169], who achieved a viability reduction of approximately 0.5 log_10_, suggesting that this bacterium is extremely difficult to eliminate.

More examples of the use of 5-ALA-mediated aPDI toward Gram-negative bacteria are shown in Table 3.

It should be noted that the effectiveness of photodynamic inactivation may depend on the species of bacteria and the virulence factors. It can be observed that photoinactivation of planktonic *Escherichia coli* is more effective than *Klebsiella pneumoniae* under identical killing conditions [158]. Furthermore, the elimination of resistant bacteria from planktonic species is more effective compared to those that occur in the form of biofilms [118].

The results presented in Table 2 and Table 3 show that the effectiveness of aPDI in combating pathogens depends not only on the strain and its growth form (plankton; biofilm), but also on the externally administered 5-ALA concentration and the wavelength and dose of light used. Careful analysis of these data highlighted several important issues that require discussion. It should be emphasized that the concentration of 5-ALA (externally delivered to cells) must ensure effective intracellular porphyrin synthesis and remain at a non-toxic level relative to cells that are not the target of this treatment. Unfortunately, the problem of cytotoxicity of this amino acid has not been thoroughly investigated. Chu et al. [170] evaluated the cytotoxic effect of 5-ALA and its hexyl ester on human lymphocytes. These authors observed that lymphocytes after incubation with 5-ALA at a concentration of 0.75 mM (in the dark) were characterized by significant DNA damage.

The results obtained by Chu et showed that this amino acid can be harmful, and this aspect should be taken into account when developing a protocol for the photodynamic destruction of microorganisms. It seems that research should be carried out in this area to demonstrate that human cells are particularly sensitive to the toxic effects of 5-ALA.

Many authors using 5-ALA-aPDI to remove bacterial pathogens do not provide a criterion for selecting the concentration of 5-ALA supplied to cells exogenously. This is extremely important because it is known that the concentration of intracellular 5-ALA is a critical parameter of the metabolism of this amino acid. For example, 5-ALA at concentrations ranging from 0.05 mM [147] to 10 mM [146] for effective photo-elimination of *Staphylococcus aureus* were used (Table 2). In the case of *Listeria monocytogenes*, 5-ALA concentrations ranged from 7.5 mM to 10 mM.

A wide range of 5-ALA concentrations were also used to effectively destroy Gram-negative bacteria (Table 3). For example, 5-ALA has been applied at concentrations ranging from 0.1 mM [119] to 40 mM [148] in the photodynamic destruction of *Escherichia coli*.

Similar discrepancies concern the preincubation time of bacterial cells with 5-ALA. As seen in Table 2, the preincubation time of staphylococcal cells with 5-ALA ranged from 1 h [148] to 4 h [147]. In the case of enterococci, an extremely short preincubation time was used (only 30 s). However, attention should be paid to the very high concentration of 5-ALA acid, which was 62.5 mM and 125 mM for *Streptococcus sobrinus* and *Streptococcus mutans*, respectively [153].

It seems that researchers arbitrarily chose the time of preincubation of bacteria with 5-ALA, without analyzing the direct relationship between the time of cell contact with this amino acid and the production of porphyrins, which are actual photosensitizers.

Two other criteria that should also be taken into account are the light source and the light dose, which are key to photodynamic destruction; moreover, these parameters should be selected individually for a given photosensitizer not only based on the absorption spectra [16]. These parameters must be standardized, allowing an effective in vivo treatment approach to be developed.

As can be seen in Table 2 and Table 3, various light sources were used in 5-ALA-aPDI studies, including halogen lamps, xenon lamps, and LEDs [118,148,171]. The UV-vis absorption spectra of protoporphyrin IX are known to show a distinct Soret band located around 400 nm, as well as weaker Q bands (628 nm, 573 nm, 537 nm, 502 nm) [172]. It therefore appears that blue light (around 400 nm) should be used as the first choice of wavelength. This problem was analyzed by Dan et al. [173] (in relation to the destruction of cancer cells) and it has been shown that although 405 nm can maximize the effectiveness of PpIX fluorescence excitation, the effective penetration depth of this light was limited (less than 1 mm). The authors concluded that this poor light penetration requires the use of a high-power light source.

Excitation of PpIX with 630 nm light was applied as an alternative strategy that had greater advantages in terms of excitation depth [174].

Very interesting results were shown by Myrzakhmetov et al. [175]. These authors proved that changing the polarity of the medium drastically affects the absorption spectrum of PpIX in the UV-visible range. In the literature, it is often claimed that PpIX should be excited at 630 nm in vitro or in vivo. This excitation wavelength is based on the absorption spectrum in ethanol. In other aqueous media (e.g., PBS buffer), which is an aqueous medium more similar to physiological media, the Q band is located at 641 nm.

An important aspect of the effective elimination of bacterial pathogens is also the dose of light. As seen in Table 2 and Table 3, the light doses used by the researchers varied significantly. Too small doses may reduce the effectiveness of aPDI, while too large doses result in false results. For example, overheating the layer of the bacterial cell can inappropriately increase the effectiveness of aPDI. Radiation exposure must be optimized to exclude factors that may negatively affect therapy results. At this point, it is worth considering whether, instead of one long-term exposure, it would be better to perform several shorter sessions.

**Table 3:** Applications of 5-ALA-mediated PDI against Gram-negative bacteria.

Bacterial Strain Gram (-)	Concentration of 5-ALA	Light Dose	Light Source	Pre-Incubation Time with 5-ALA	Viability Reduction	Ref.
*Escherichia coli* (clinical isolate) Planktonic	40.00 mM	142 Jcm^−2^	ELH tungsten-halogen GE Quartzline lamps 25.2 mWcm^−2^	4 h	Not responsive to aPDI	[148]
*Escherichia coli* (clinical isolate) Biofilm	40.00 mM	142 Jcm^−2^	ELH tungsten-halogen GE Quartzline lamps 25.2 mWcm^−2^	4 h	Not responsive to aPDI	[148]
*Pseudomonas aeruginosa* ATCC 27853 Planktonic	40.00 mM	142 Jcm^−2^	ELH tungsten-halogen GE Quartzline lamps 25.2 mWcm^−2^	4 h	4.00 log_10_ of CFUcm^−3^	[148]
*Pseudomonas aeruginosa* ATCC 27853 Biofilm	40.00 mM	142 Jcm^−2^	ELH tungsten-halogen GE Quartzline lamps 25.2 mWcm^−2^	4 h	Not responsive to aPDI	[148]
*Escherichia coli* K-12 Planktonic	0.10 mM	120 Jcm^−2^	400 W-halogen lamp	4 h	3.31 log_10_ of CFUcm^−3^	[119]
*Escherichia coli* K-12 Planktonic	1.00 mM	120 Jcm^−2^	400 W-halogen lamp	4 h	4.30 log_10_ of CFUcm^−3^	[119]
*Pseudomonas aeruginosa* ATCC 2338 biofilm	0.50%	9 Jcm^−2^	Light-emitting diode 410 nm	4 h	5.00 log_10_ of CFUcm^−3^	[167]
*Pseudomonas aeruginosa* ATCC 27853 Biofilm	1.408 M	54 Jcm^−2^	Light-emitting diode 630 nm	0.5 h	No growth of bacteria after 14 days of wound healing	[176]
*Pseudomonas aeruginosa* PAO1 Biofilm	20.00 mM	240 Jcm^−2^	Light-emitting diode 630 nm	1 h	Complete inactivation	[168]
*Pseudomonas aeruginosa* PAO1 Planktonic	10.00 mM 7.50 mM	240 Jcm^−2^ 360 Jcm^−2^	Light-emitting diode 630 nm	1 h	Complete inactivation	[168]
*Salmonella enterica* DS88 planktonic	0.50 mM	10 Jcm^−2^ 20 Jcm^−2^	Light-emitting diode 400 nm	4 h	Approximately 2.2 log_10_ of CFUcm^−3^ Approximately 1.7 log_10_ of CFUcm^−3^	[171]
*Salmonella enterica* DS88 Planktonic	0.50 mM	10 Jcm^−2^ 20 Jcm^−2^	Light-emitting diode 400 nm	20 h	Approximately 1.75 log_10_ of CFUcm^−3^ Approximately 1.35 log_10_ of CFUcm^−3^	[171]
*Klebsiella pneumoniae* ATCC 700603 Planktonic	10.00 mM	360 Jcm^−2^	150 W xenon lamp A wavelength range between 400 and 780 nm	4 h	3.68 log_10_ of CFUcm^−3^	[118]
*Klebsiella pneumoniae* ATCC 700603 Biofilm	10.00 mM	360 Jcm^−2^	150 W xenon lamp A wavelength range between 400 and 780 nm	4 h	3.09 log_10_ of CFUcm^−3^	[118]
*Klebsiella pneumoniae* (non-ESBL-producing clinical isolate) Planktonic	10.00 mM	360 Jcm^−2^	150 W xenon lamp A wavelength range between 400 and 780 nm	4 h	3.17 log_10_ of CFUcm^−3^	[118]
*Klebsiella pneumoniae* (non-ESBL-producing clinical isolate) Biofilm	10.00 mM	360 Jcm^−2^	150 W xenon lamp A wavelength range between 400 and 780 nm	4 h	1.92 log_10_ of CFUcm^−3^	[118]
*Klebsiella pneumoniae* (ESBL-producing clinical isolate) Planktonic	10.00 mM	360 Jcm^−2^	150 W xenon lamp A wavelength range between 400 and 780 nm	4 h	3.20 log_10_ of CFUcm^−3^	[118]
*Klebsiella pneumoniae* (ESBL-producing clinical isolate) Biofilm	10.00 mM	360 Jcm^−2^	150 W xenon lamp A wavelength range between 400 and 780 nm	4 h	2.28 log_10_ of CFUcm^−3^	[118]
*Escherichia coli* IQ0245 Planktonic	50.00 µgmL^−1^	80 Jcm^−2^	Light-emitting diode 405 nm	0.5 h	5.00 log_10_ of CFUcm^−3^	[158]
*Klebsiella pneumoniae* IQ0035 Planktonic	50.00 µgmL^−1^	80 Jcm^−2^	Light-emitting diode 405 nm	0.5 h	3.00 log_10_ of CFUcm^−3^	[158]
*Acinetobacter baumannii* ATCC 19606 Planktonic	1.25 mM	32 Jcm^−2^	Diode laser 405 nm	4 h	Lethal effect	[163]
*Acinetobacter baumannii* ATCC 19606 Planktonic	1.25 mM	102 Jcm^−2^	Diode laser 635 nm	4 h	Lethal effect	[163]

### 4.3. 5-ALA Mediated PDI of Viruses, Fungi, Yeasts, and Parasites

The continuous development of aPDI has inspired scientists to investigate the effectiveness of this technique against viruses. Most in vitro virus research has focused on the application of sterilization methods of blood [177]. Most clinical applications of PDI for localized infections have focused on viral etiology lesions. There is great interest in the use of 5-ALA in the photodynamic inactivation of viruses. For example, Zhang et al. [170] performed PDI treatment (20 min of irradiation, λ = 635 nm, energy fluence of 50–60 Jcm^−2^) in recalcitrant facial flat warts with topical 10% 5-ALA. They reported that 24 weeks after the start of therapy, seven of fifteen patients showed a complete resolution of the lesions, while only one patient showed a poor response to this form of therapy (<30% resolution of the lesions). These results demonstrated that the topically used 10% 5-ALA-aPDI is an effective and safe treatment for the HPV virus [178]. Similar research was conducted by Chen et al. [179], who showed that persistent HPV infections can be effectively treated with blue light (450 nm) in the presence of 5-ALA.

Every year, fungi are responsible for more than one billion diseases around the world [180]. The increasing number of fungal infections has led to the search for new alternative methods to combat them. For example, Lan et al. [181] described the effectiveness of 5-ALA use in PDI against *Trichosporon asahii* [181]. A patient with trichosporonosis was successfully cured.

5-ALA and light combinations have also been reported to kill human pathogenic parasites. Souza et al. [182] found that photodynamic inactivation with 5-ALA reduced parasite load in mice infected with *Leishmania braziliensis.*

### 4.4. Applications of 5-ALA in Chronic Infectious Leg Ulcers

In recent years, 5-ALA has been shown to be extremely effective in the treatment of chronic infectious leg ulcers. Lin et al. [183] demonstrated the success of aPDI in healing chronic infected leg ulcers in three patients who were disease-free for more than 29 months. 5-Aminolevulinic acid was dissolved in 0.9% saline to obtain a concentration of the studied amino acid of 2%. Then, a layer of gauze saturated with 2–3 mL of sterile solution was applied to the ulcers, which were then occluded for 6 h with an adhesive membrane and aluminum foil to avoid light. The ulcers were irradiated with a lamp (wavelength 560 to 780 nm) at a light dose of 20 Jcm^−2^. The authors emphasized that the patients tolerated aPDI well, except for slight tingling during irradiation. Importantly, after one treatment in each patient, it was not possible to isolate any bacteria from the ulcers treated with aPDI. To further investigate how aPDI alters the wound microenvironment through interleukin-6 (IL-6), a key cytokine involved in wound healing, conditioned medium with and without aPDI was added to a human basal cell carcinoma cell line. The results obtained showed that the conditioned medium improved cell migration, but this effect was blocked by the IL-6 monoclonal antibody. These findings supported the hypothesis that aPDI could alter the wound microenvironment at least partially through the IL-6 signaling pathway [184].

**Figure 5:**
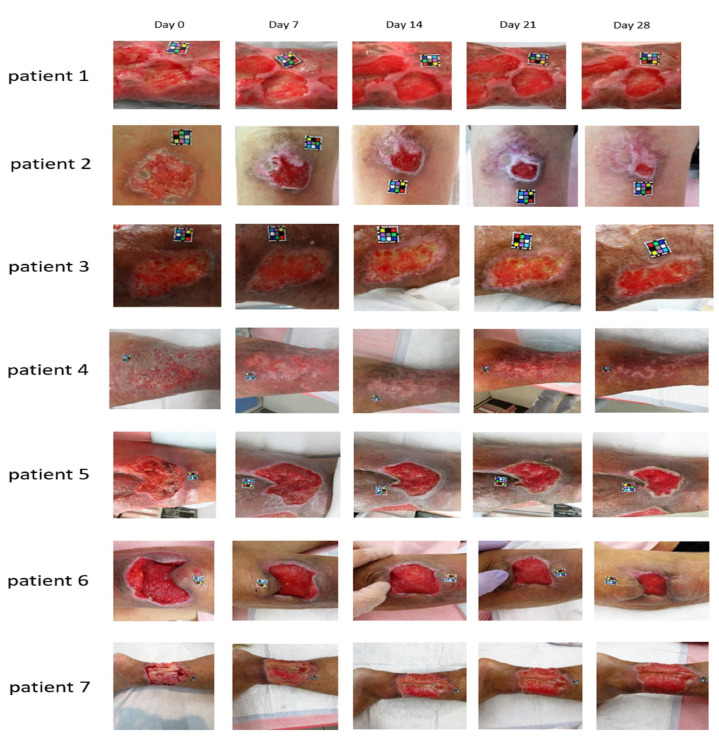
The clinical course of ulcers. A reduction in the ulcer area or an improvement in granulation was observed in seven patients. Result of days 0, 7, 14, 21, and 28 are shown. The squares in the figure represent the reference sticker assigned to each patient. Taken from [185]; All Translational Biophotonics articles are published under a Creative Commons Attribution License.

Another study conducted between March 2018 and March 2019 enrolled patients with skin ulcers caused by strains of *Staphylococcus aureus* MRSA and *Pseudomonas aeruginosa* PA [185]. The photosensitizer was prepared at a final concentration of 0.5% ALA-HCl and 0.005% EDTA-2Na dissolved in Macrogol ointment. The LED light source was Aladuck LS-DLED^®^ SBI Pharmaceuticals Co.; Tokyo, Japan (wavelength: 410 nm). On the first day of the procedure, 5-ALA ointment was applied to the affected areas. The next day, the affected area was irradiated with LED light with an energy of 10 Jcm^−2^. Gauze was used during irradiation to protect the surrounding skin from light. After the radiation was completed, 5-ALA ointment was applied to the affected area, covered with gauze, and a dressing was applied. These procedures were repeated once a day until the site of infection disappeared, and the condition of the wound improved. The authors reported that a low concentration of 5-ALA (0.5% in this study) and a short wavelength (410 nm in this study) avoided pain or adverse events during or after aPDI, and that the technique was considered safe to use in the following days of treatment. The area was reduced in all patients within 4 weeks after starting the study. Six out of seven patients showed a clear trend for the ulcer area to decrease to 60% of the value measured at baseline (Figure 5).

It should be emphasized that side effects seem to be an important aspect of this therapy. For example, Lei et al. [186] used aPDI to treat patients with *Pseudomonas aeruginosa*-infected skin ulcers once a week for 2 weeks. These authors applied 5-ALA at a concentration of 20% and a red light with a wavelength of 630 nm. All patients reported pain during treatment and swelling and redness around the skin ulcer for 1 to 3 days after treatment.

Nakano et al. [187] performed aPDI in Japanese patients with actinic keratosis by applying 20% 5-ALA for 4 h and then irradiating it with 630 nm light three times at 7-day intervals. In this case, all patients also experienced different types of pain during this treatment. Furthermore, erythema was observed, with subsequent pigmentation, although scarring and discoloration disappeared after 12 months.

Similarly, in a pilot study conducted by Krupka et al. [188] moderate pain and moderate local swelling and erythema were observed as side effects in chronic leg ulcers irradiated with 20% ALA for 4 h (630 nm).

Due to the significant medical and social problem of chronic leg ulcers, the use of photodynamic therapy in their treatment seems to be justified, especially due to the need for long-term antibiotic therapy resulting in the emergence of antibiotic-resistant pathogens.

### 4.5. 5-ALA-Mediated Photodynamic Therapy in Endodontics

The use of 5-ALA-mediated photodynamic therapy in endodontics is a topic widely discussed in the literature [189,190]. Endodontic infections are polymicrobial, but in the case of primary infections, anaerobic bacteria absolutely dominate the microbiota. There are various microorganisms responsible for intra-radicular and extra-radicular infections, and organisms involved in persistent infections. The dominant microorganisms causing these infections are anaerobic bacteria, such as *Actinomyces* spp., *Propionibacterium propionicum*, *Treponema* spp., *Porphyromonas endodontalis*, *Porphyromonas gingivalis*, *Treponema* *forsythia*, *Prevotella* spp., and *Fusobacterium nucleus* [191].

The effectiveness of photodynamic inactivation in endodontics is still controversial. The use of sodium hypochlorite (0.5%) and NaOCl (2.5%) as a biocidal method has been shown to be as effective as aPDI [192,193]. It seems that the effectiveness of removing pathogens from root canals increased when combined with chemo-mechanical treatment (sodium hypochlorite and EDTA irrigation) and photodynamic treatment based on the 5-aminolevulinic acid used [194,195,196]. It should be taken into account that the success of photodynamic protocols is related to the presence of sufficient oxygen. Therefore, the small amount of oxygen present in the root canal system and the dentin tubules can constitute a limit to the photodynamic efficiency in endodontics.

### 4.6. The Combined Effect of 5-ALA-aPDI with Other Agents

The effectiveness of aPDT through 5-ALA can be made extremely effective by combining this method with antimicrobial drugs. Antibiotics and antifungals combined with photodynamic microbial inactivation have been shown to have advantages in terms of shorter treatment durations, lower doses, less toxicity, better patient compliance, and a lower chance of drug resistance. The mechanism behind this improvement in the effectiveness of photodynamic therapy may be that aPDI damages the cell wall or cell membrane of the microorganism, thus allowing better penetration of the antimicrobial drug [197,198].

For example, Zhang et al. [146] clearly demonstrated that the combination of 5-ALA-aPDI and netilmicin, vancomycin, or cefaclor could produce a combined effect on the biofilm of *Staphylococcus aureus* in a strain-dependent manner.

Lan et al. [181] evaluated the inhibitory effect of 5-ALA-aPDI in combination with antifungal drugs on *Trichosporon* spp. The results obtained showed that the antifungal effect of 5-ALA-aPDI was dependent on the concentration and dose of 5-ALA, and that the destruction of the biofilm was more effective as a result of the combined action of light and itraconazole.

Another study evaluated the performance of photodynamic inactivation using 630 nm and 880-nm LEDs for killing *Staphylococcus aureus* and *Pseudomonas aeruginosa* bacteria in a dual species biofilm in the Lubbock chronic wound biofilm (LCWB) model. It has been shown that for effective treatment of microbial proliferation in LCWB, the combined action of graphene oxide and the repeated action of photodynamic therapy with 5-ALA should be used [199]. 

## 5. Other Applications of 5-ALA

**Figure 6:**
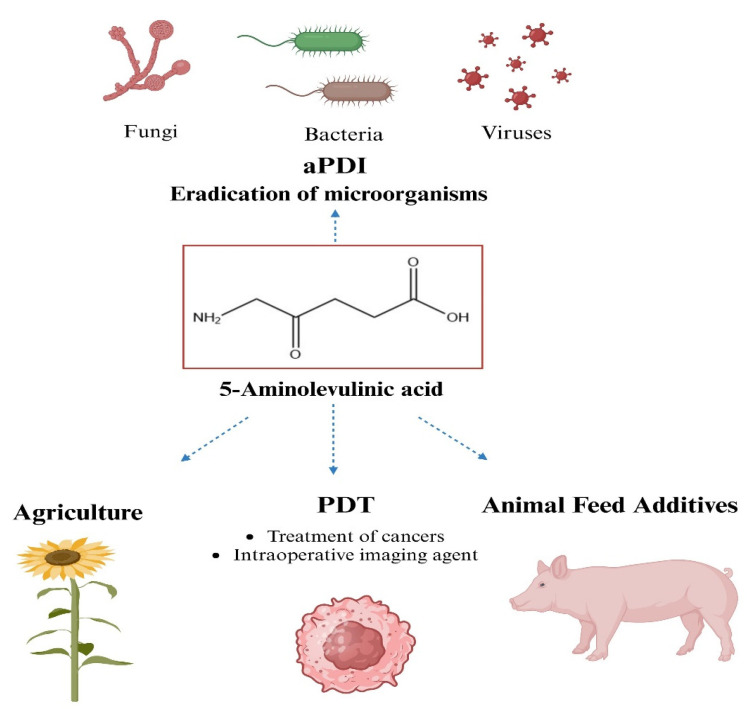
Applications of 5-ALA.

Throughout the following decades, 5-ALA became an attractive topic for research. The popularity of this chemical is explained by its wide range of applications (Figure 6).

This amino acid is used in the cultivation of plants as a growth regulator, in medical and cosmetic areas, and as an animal feed additive with great potential [200,201,202]. For example, exogenous treatment of plants with 5-ALA has been demonstrated in recent studies to improve tolerance to environmental stress by triggering molecular and physiological defense responses, representing an effective approach to alleviating abiotic factors in plants [203].

Great attention is also focused on 5-ALA, which, as one of the components of the heme biosynthesis pathway, has the potential to support animal production [80]. Overall, it can be established that 5-ALA is a promising animal feed supplement that can enhance iron status and immunological response [20].

## 6. Conclusions

The interest of researchers in the possibility of using antimicrobial photodynamic therapy is undoubtedly due to the simple treatment plan: a local bacterial infection is treated with a photo-active, non-toxic chemical called a photosensitizer (PS). This is followed by a brief interaction of this photosensitizer with the infected area. In the next step, the target is exposed to visible light of the appropriate wavelength, which causes the PS to excite and enter the active triplet state. A large amount of ROS is produced when energy or electrons are transferred to molecular oxygen. It should be noted that the effective concentration of photosensitizers in target cells may be low because a single molecule of a photoactive substance can photo-catalyze many units of reactive oxygen. Furthermore, the diffusion of singlet oxygen is limited to 30 nm, which means that cell damage can only occur in close proximity to the photosensitizer [204].

5-ALA is a naturally occurring compound involved in physiological cellular metabolism and is the basic precursor for the biosynthesis of heme groups. It has been clearly demonstrated that selective accumulation of protoporphyrin IX can be observed in cells that are characterized by a high metabolic turnover, such as tumors, macrophages, and bacteria, after exogenous administration of 5-ALA.

Since its initial approval in 1999, the US FDA has approved five 5-ALA-based drugs [1,205]. These drugs are approved not only in the US but also in many other countries. They play an important role in the treatment of some precancerous lesions and cancers. It has been shown that their applications include the effective and rapid removal of abnormally proliferative tissues, but also facilitate the detection of tumors based on the selective 5-ALA-induced accumulation of PpIX in target tissues.

In this review, past and recent (up to the end of 2023) in vitro experiments that examined the feasibility of using 5-ALA for light-induced inactivation of bacterial cells are summarized. The most important conclusion of these data concerns the lack of a standardized protocol for testing the effectiveness of aPDI against microorganisms. Most studies do not clearly demonstrate the relationship between the concentration of 5-ALA delivered to cells and the level of accumulated intracellular photosensitizers. Researchers’ attention should be focused on this problem because while in cancer cells the transformation of 5-ALA to photo-active protoporphyrin IX takes place along the same metabolic pathways, in the case of prokaryotic cells the formation of photo-active porphyrins may involve more than one heme pathway.

The dark cytotoxicity of this amino acid is also not taken into account. The toxicity of 5-ALA toward cells that are not the target of light-induced therapy is a factor that limits the clinical applications of this treatment procedure. A few years ago, it was shown that the key factor in suspending light-based treatment was the pain reported by patients [206,207]. Modification of the treatment protocol consisted of lowering the 5-ALA concentration and increasing the light not only reduced pain but had an acceptable therapeutic effect in severe acne vulgaris [208], genital warts [209] and cSCC [210]. There is a clear need to conduct research in this area relating to photo-control of the development of pathogens.

Although 5-ALA is a natural amino acid, its disadvantages, such as low lipophilicity, low stability, and poor bioavailability, significantly reduce the clinical effectiveness of this prodrug. Nanotechnology is expected to solve these problems and enable enhanced action of this drug, controlled release, and excellent targeting of pathogenic cells. Unfortunately, the data available in the literature have shown that progress in the use of various 5-ALA-based nanomedicines for the removal of pathogens is insufficient. The innovative design of biocompatible and degradable nanosystems is necessary to ensure greater opportunities to reach the clinical stage.

The weak ability of 5-ALA to cross biological barriers has led to the introduction of more lipophilic derivatives, such as methyl aminolevulinate or hexyl aminolevulinate, which display improved capacity to reach the cytoplasm. This is worth noting that in Gram-negative bacteria, the double membrane prevents the penetration of exogenous molecules, while porins, which are transmembrane channels, allow only small hydrophilic molecules, usually nutrients, to enter the cells. Thus, the entrance of 5-ALA into Gram-negative bacteria is not problematic, as demonstrated by the low concentration of 5-ALA necessary to effectively inactivate these pathogens.

Given its excellent antibacterial properties of 5-ALA-aPDI and its role in promoting wound healing and endodontics, the development of effective clinical regimens for the treatment of bacterial infections could reduce the use of antibiotics, thus slowing the emergence of drug-resistant strains. However, optimization of the photodynamic microbial inactivation protocol(s) is/are required in terms of 5-ALA concentration and type/dose of light, as well as strategies to enhance the 5-ALA-aPDI response, which may improve efficacy and therapeutic outcomes.
